# Enhancement of the Gene Targeting Efficiency of Non-Conventional Yeasts by Increasing Genetic Redundancy

**DOI:** 10.1371/journal.pone.0057952

**Published:** 2013-03-07

**Authors:** Zao Chen, Hongbing Sun, Pengfei Li, Ning He, Taicheng Zhu, Yin Li

**Affiliations:** 1 Department of Chemical and Biochemical Engineering, College of Chemistry and Chemical Engineering, Xiamen University, Xiamen, People’s Republic of China; 2 Institute of Microbiology, Chinese Academy of Sciences, Beijing, People’s Republic of China; 3 College of Life Science and Agriculture, Hainan University, Haikou, People’s Republic of China; University of Strasbourg, France

## Abstract

In contrast to model yeasts, gene targeting efficiencies of non-conventional yeasts are usually low, which greatly limits the research and applications of these organisms. In this study, we aimed to enhance the gene targeting efficiency of non-conventional yeasts by improving the fitness of mutant strains, particularly by increasing the genetic redundancy of host cells. To demonstrate this process, *OCH1* gene deletion in *Pichia pastoris* was performed. Extra copies of the *OCH1* gene on a helper plasmid were provided for the *P. pastoris* GS115 strain before the native *OCH1* gene in the genomic DNA was knocked out. The redundancy in *OCH1* gene significantly eliminated the growth defects of the *och1* mutant and increased the deletion efficiency of the *OCH1* gene by two orders of magnitude with the same length of homologous flanks. The same strategy was used to delete the *KU70* and *SGS1* genes. The targeting efficiencies of *KU70 and SGS1* were increased by 1- and 23-fold, respectively. Therefore, this study provided an efficient strategy for the deletion of “stubborn” genes in non-conventional yeasts. This study further showed that cellular fitness is potentially an important factor that can limit the efficiency of gene targeting.

## Introduction

Gene targeting (e.g., targeted gene replacement) is one of the main molecular tools used in yeast science, which helps in understanding gene functions and interactions as well as cellular and molecular processes in yeasts [1]. Model yeasts, such as *Saccharymyces cerevisiae* and *Schizosaccharomyces pombe*, have very efficient gene targeting systems. For instance, disruption cassettes with short flanking regions that range from 30 bp to 50 bp can integrate with high efficiencies via homologous integration at the correct genomic locus (routinely >70%) in *S. cerevisiae* [2]. In contrast, the gene targeting efficiencies of various “non-conventional” yeasts [3], [4], such as *Pichia pastoris*, *Hansenula polymorpha, Yarrowia lipolytica, Pichia stipitis* and *Kluyveromyces lactis* can be extremely low (usually <1%) with the same length of flanking regions. Accordingly, homologous arms varying from 200 bp to approximately 2000 bp are usually required to ensure efficient gene replacement in non-conventional yeasts [1]. Nevertheless, for some “stubborn” genes, the probability of obtaining the desired gene replacement event is so low that transformation and/or screening procedures have to be iteratively performed, which is laborious and time-consuming. Therefore, the low targeting efficiencies of non-conventional yeasts greatly limits the researches and applications of these industrially important microorganisms.

To improve the targeting efficiencies of non-conventional yeasts, molecular mechanisms of gene targeting should be understood. Yeasts have the homologous recombination (HR) pathway and the non-homologous end-joining (NHEJ) pathway. The HR pathway, which depends on the Rad52 epistatic group, is responsible for the targeted integration of DNA [5]. Integration can also be mediated randomly via the NHEJ pathway, which depends on the Ku70/Ku80 protein complex [6]. When foreign DNAs are transformed into cells, they are competed by these two recombinant pathways. Therefore, efficient gene targeting is determined by the relative strength of the HR pathway compared with that of the NHEJ pathway. The bias, which favors the NHEJ pathway in non-conventional yeasts, determines their low targeting efficiencies [1].

However, NHEJ pathway bias is not the only factor that contributes to the low targeting efficiency of non-conventional yeasts. The efficiency of homologous integration in strains with the same genetic background can be very locus specific. For example, the disruption of *ARG1, ARG2, ARG3, HIS1, HIS2, HIS5,* and *HIS6* in *P. pastoris* GS115 strain with flanking arms that range from 200 bp to 900 bp is considerably efficient at frequencies of 44% to 90% [7]. However, the deletion of *PIM* [8] and *OCH1* [9] from the same strain that contains both flanking arms that were longer than 1 kb occurred at a frequency of <1%. To date, this locus specific phenomenon is not well understood. One explanation is the presence of “hotspot” regions for yeast genome, in which the underlying mechanism still remains unclear [10]. Another, which is often overlooked in previous studies, may be the loss of function effect. Several non-conventional yeasts such as *P. pastoris*, *H. polymorpha*, *Y. lipolytica*, and *P. stipitis* are predominantly haploid [11], [12]. Thus, the knockout of genes with important physiological functions often means great loss of cellular fitness, which would lead to a delayed or failed appearance of correct disruptants. Therefore, the calculated frequency of correct gene targeting by colony counting is likely lower than the actual frequency of gene targeting events that happen genetically because of the unformed colonies.

**Figure 1:**
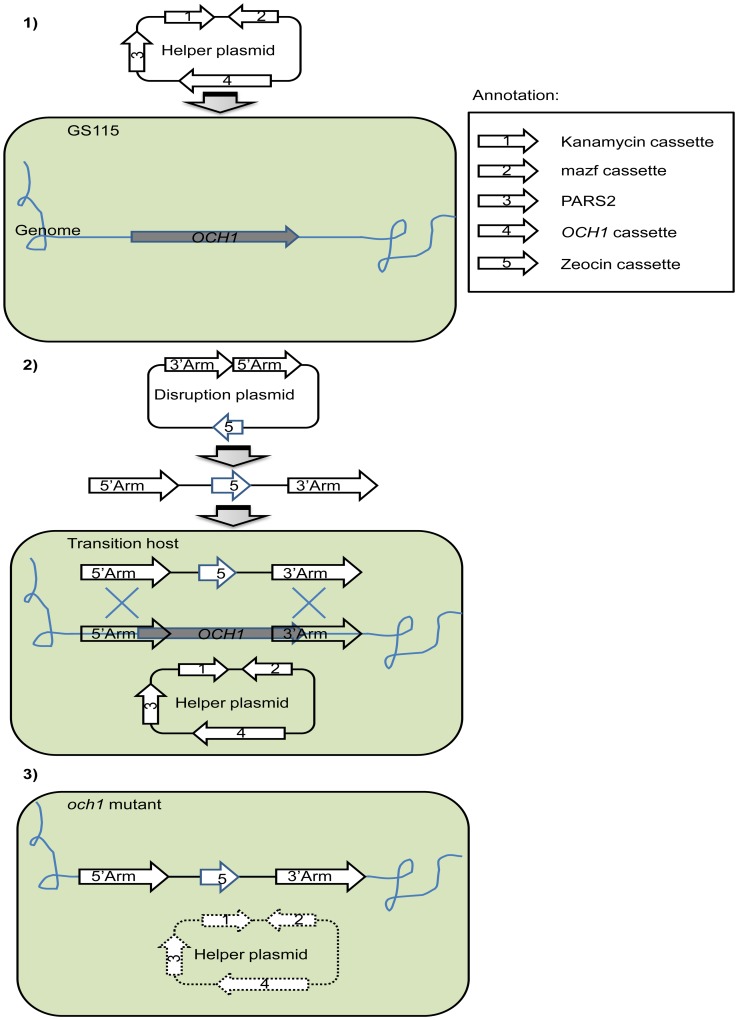
Schematic representation of the new strategy for efficient gene targeting. Take deletion of *OCH1* gene as an example. As follows, it’s a three-step process: 1) Generation of the transition host. The helper plasmid, which has the cloned *Pichia pastoris OCH1* gene placed under the control of the glyceraldehyde-3-phosphate dehydrogenase (GAP) promoter, was transformed into *P. pastoris* GS115 strain, thereby generating the transition host for *OCH1* gene deletion. The helper plasmid can be maintained within the host cell because of the presence of an autonomous replication sequence (PARS2). 2) Gene disruption. The disruption plasmid for *OCH1*, which contains the 5′ and 3′ flanking regions of the *OCH1* gene, was linearized and transformed into the transition host for gene deletion. 3) Elimination of the helper plasmid. After the chromosomal *OCH1* gene was successfully deleted, cells were streaked on an agar plate with methanol to induce the expression of *E. coli mazf* gene (encoding MazF). MazF toxicity causes a strong selection pressure on the streaked strains and induces the loss of the helper plasmids.

To validate this assumption as well as provide an efficient solution to delete “stubborn” genes in haploid non-conventional yeasts, in this study, we proposed a new strategy (Fig. 1) for enhancement of gene targeting efficiency by improving cellular fitness of mutant cells, specifically by increasing the genetic redundancy of host cells. To achieve this goal, the targeted gene was cloned into an expression vector (helper plasmid), and transformed into yeast cells to generate the transition host. Targeted gene disruption was then applied in the transition host by transformation with the disruption plasmid. After gene deletion in the genome was successfully validated, the helper plasmid can be easily removed. *P. pastoris*, a methylotrophic yeast, was used as a model in this study for two reasons: 1) the efficiency of targeted gene replacement in *P. pastoris* is very low, which is estimated to occur at a frequency of 0.1% when the total length of the target fragments is 500 bp [13]; and 2) *P. pastoris* is of great industrial importance. It is by far one of the most often used yeast species in the production of recombinant proteins [14]. The *OCH1* gene of *P. pastoris* was chosen as an illustrative example because its deletion procedure by double homologous recombination is notoriously inefficient [9], [15]. To demonstrate the effectiveness of this strategy, the deletion of two other genes, namely, *SGS1* and *KU70*, was also assessed using the same method.

## Materials and Methods

### Strains, Plasmids, and Oligonucleotides

A complete list of strains and plasmids is presented in Table 1. Oligonucleotide synthesis (Table S1) and DNA sequencing were performed at the Shanghai Sangon Biological Engineering Technology and Service Co. (Beijing, China).

**Table 1:** Strains and plasmids used in this work.

Strains	Characteristics	Reference
*E.coli* DH5α	General cloning host strain	Takara
***P. pastoris***		
GS115	*his4* mutant	Invitrogen
GS-tranOCH1	GS115 carrying pGKARSmazf-OCH1	This work
GS-tranSGS1	GS115 carrying pGKARSmazf-SGS1	This work
GS-tranKU70	GS115 carrying pGKARSmazf-KU70	This work
KO24#	Reconstructed by single crossover	This work
GS-tranOCH1-ΔOCH1	*och1* mutant carrying pGKARSmazf-OCH1	This work
GS -ΔOCH1	*och1* mutant	This work
**Plasmids**		
pUG6	Base vector for constructing pZeoloxp	Invitrogen
pUG-zeoloxp	Vector for constructing pZeoloxp	This work
pZeoloxp	Vector for constructing disruption plasmids	This work
pZeoloxp-OCH1	pZeoloxp based vector for deletion of *OCH1*	This work
pZeoloxp-SGS1	pZeoloxp based vector for deletion of *SGS1*	This work
pZeoloxp-KU70	pZeoloxp based vector for deletion of *KU70*	This work
pGAPZB	Vector for constitutive protein expression	Invitrogen
pPIC9K	Vector used for amplification of Kan^R^ cassette	Invitrogen
pGAPKB	pGAPZB based vector replacing Zeo^R^ into Kan^R^	This work
pGKARS	pGAPKB based vector carrying an PARS2	This work
pPICZA	Vector for amplification of Zeo^R^, Amp^R^ and Ori	Invitrogen
pGKARS-OCH1	Vector for constitutive expression of ORF *OCH1*	This work
pGKARS-SGS1	Vector for constitutive expression of ORF *SGS1*	This work
pGKARS-KU70	Vector for constitutive expression of ORF *KU70*	This work
pGKARSmazf-OCH1	pGKARS-OCH1 based vector carrying a *mazf* gene	This work
pGKARSmazf-SGS1	pGKARS-SGS1 based vector carrying a *mazf* gene	This work
pGKARSmazf-KU70	pGKARS-KU70 based vector carrying a *mazf* gene	This work

**Abbreviations:** Amp^R^, ampicillin resistance; Kan^R^, kanamycin resistance; Zeo^R^, zeocin resistance.

### Media and Growth Conditions

*Escherichia coli* DH5α strain was grown at 37°C in LLB broth (10 g L^–1^ of tryptone, 5 g L^–1^ of yeast extract, 5 g L^–1^ of NaCl) or LB medium (10 g L^–1^ of tryptone, 5 g L^–1^ of yeast extract, 10 g L^–1^ of NaCl). *P. pastoris* was cultivated aerobically at 30°C in yeast extract peptone dextrose (YPD) medium (10 g L^–1^ of yeast extract, 20 g L^–1^ of peptone, 20 g L^–1^ of glucose). Antibiotics were added at the following concentrations: 100 mg L^–1^ of ampicillin, 50 mg L^–1^ of kanamycin, and 25 mg L^–1^ of Zeocin (Invitrogen) for *E. coli*; 500 mg L^–1^ of geneticin (G418, Invitrogen) and 50 mg L^–1^ of Zeocin for *P. pastoris*.

### Construction of Disruption Plasmids

pZeoloxp, the base vector used to construct all of the other disruption plasmids, was constructed based on the following procedures. Zeocin resistance cassette was amplified from pPICZA with P_zeo1_/P_zeo2_, digested with *Sac*I and *Bgl*II, and cloned into pUG6 to replace the kanMX cassette between the loxP sites, thereby producing the pUG-zeoloxp plasmid. The origin of replication (ori), which was amplified from pPICZA by polymerase chain reaction (PCR) with P_ori1_/P_ori2_ primer pair and digested with *Sal*I, was inserted into the *Xho*I site of pUG-zeoloxp. The DNA fragment with ori and ampicillin resistance gene was then excised through *Not*I digestion and religation.

**Figure 2:**
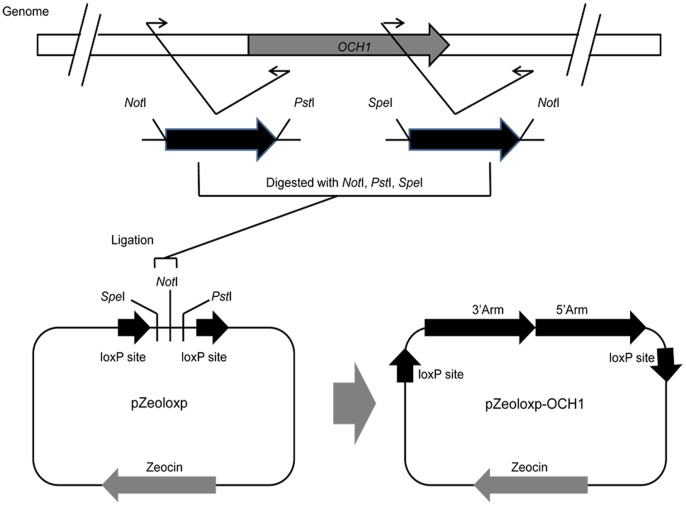
Schematic representation of the construction of the disruption plasmid. The *OCH1* gene was used as an illustrative example. Upstream and downstream homologous regions of *OCH1* were initially amplified by polymerase chain reaction from *Pichia pastoris* GS115 strain with two primer pairs. The two fragments were cut using *Spe*I, *Not*I, and *Pst*I, as well as ligated into pZeoloxp by performing a three-fragment ligation, thereby generating pZeoloxp-OCH1, which is the disruption plasmid for the *OCH1* gene.

To construct the disruption plasmid for *OCH1* deletion, the upstream and downstream homologous regions of *OCH1* were initially amplified through PCR from *P. pastoris* GS115 strain by using two primer pairs, namely, OCH1-N_5_/OCH1-N_3_ and OCH1-C_5_/OCH1-C_3_. The two fragments were cut with *Spe*I, *Not*I, and *Pst*I, ligated into pZeoloxp which was cut with *Spe*I and *Pst*I, thereby generating pZeoloxp-OCH1 (Fig. 2). The disruption plasmids used to delete the *SGS1* and *KU70* genes, namely, pZeoloxp-SGS1 and pZeoloxp-KU70 were constructed following exactly the same procedure with their corresponding primer pairs (Table S1).

### Construction of the Episomal Plasmids

pGAPZB and the kanamycin resistance gene, amplified from pPIC9K with P_kan1_/P_kan2_, were cut using *Nco*I/*Stu*I and ligated by the T4 DNA ligase to replace the original Zeocin-resistant gene, thereby generating the pGAPKB plasmid. PARS2, an autonomous replication sequence of *P. pastoris* [16], was amplified from the genomic DNA of GS115 strain with P_pars2-F_/P_pars2-R_, cut with *Bam*HI and *Bgl*II, and inserted into *Bgl*II site of pGAPKB to generate the episomal expression plasmid, pGKARS. To express *OCH1*, *SGS1*, and *KU70* in *P. pastoris* with pGKARS, their encoding genes were amplified from the GS115 genome by using their respective primer pairs (Table S1). PCR fragments were cut using *Eco*RI and *Nde*I, and then ligated into the corresponding pGKARS sites to produce the episomal expression plasmids, namely, pGKARS-OCH1, pGKARS-SGS1 and pGKARS-KU70, respectively.

**Figure 3:**
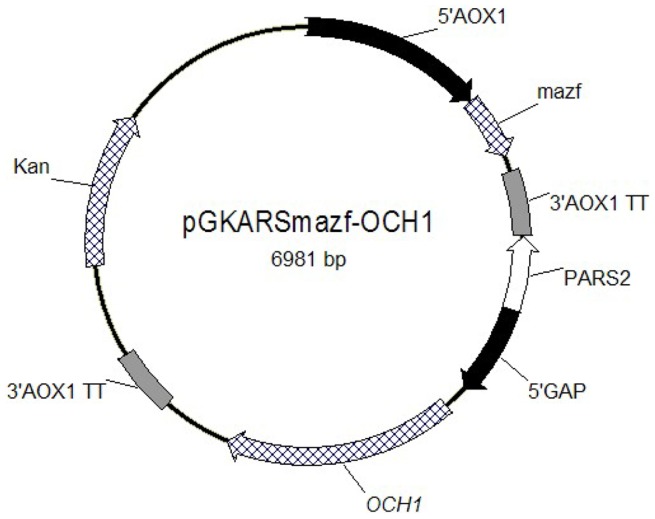
Schematic representation of the helper plasmid. pGKARSmazf-OCH1, the helper plasmid for *OCH1* gene deletion, was used as an illustrative example. This plasmid consists of three key elements, the *Pichia pastoris* autonomous replication sequence (PARS2) fragment (allows plasmid maintenance within the cells), the strong constitutive glyceraldehyde-3-phosphate dehydrogenase (GAP) promoter, and the *mazf* expression cassette (facilitates plasmid removal when necessary).

An inducible *mazf* cassette was inserted into pGKARS-OCH1 to facilitate the removal of pGKARS-OCH1 as necessary. To construct the *mazf* cassette, the *mazf* gene was amplified from *E. coli* by using the P_mazf1_/P_mazf2_ primer pair, digested with *Eco*RI and *Not*I, and ligated into the corresponding pPICZA sites to place the *mazf* gene under the control of the alcohol oxidase (AOX1) promoter. The *mazf* cassette was then excised from the resulting plasmid by *Bgl*II and *Bam*HI digestion and inserted into the *Bgl*II site of pGKARS-OCH1, thereby generating the pGKARSmazf-OCH1 plasmid (Fig. 3).

### Competent *P. pastoris* Cell Preparation and Transformation

The competent cells were prepared based on a revised version of a previously described method [17] to achieve a highly efficient transformation of *P. pastoris*. Briefly, a fresh single clone was inoculated into 100 ml YPD medium and grown overnight at 30°C by shaking at 200 rpm until the cell density reached an OD_600_ of 1∼2. The cells were pelleted and resuspended at room temperature for 30 min in 8 ml of 100 mM LiAc, 10 mM dithiothreitol, 0.6 M sorbitol, and 10 mM Tris-hydrochloride at pH 7.5. The resulting cells were then washed thrice with 2 ml to 3 ml of 1 M ice-cold sorbitol. Finally, the cells were suspended in 1 M ice-cold sorbitol and transferred to 1.5 ml microcentrifuge tubes with an aliquot of 80 µl. *P. pastoris* GS115 strain was transformed by electroporation according to the protocols outlined by Invitrogen.

### Direct Gene Disruption and Verification

The disruption plasmids (pZeoloxp-OCH1, pZeoloxp-SGS1, or pZeoloxp-KU70) were linearized by *Not*I and transformed into *P. pastoris* GS115 strain. The cells were then spread on YPD plate that contains 50 mg L^–1^ of Zeocin to screen the Zeocin-resistant transformants.

**Figure 4:**
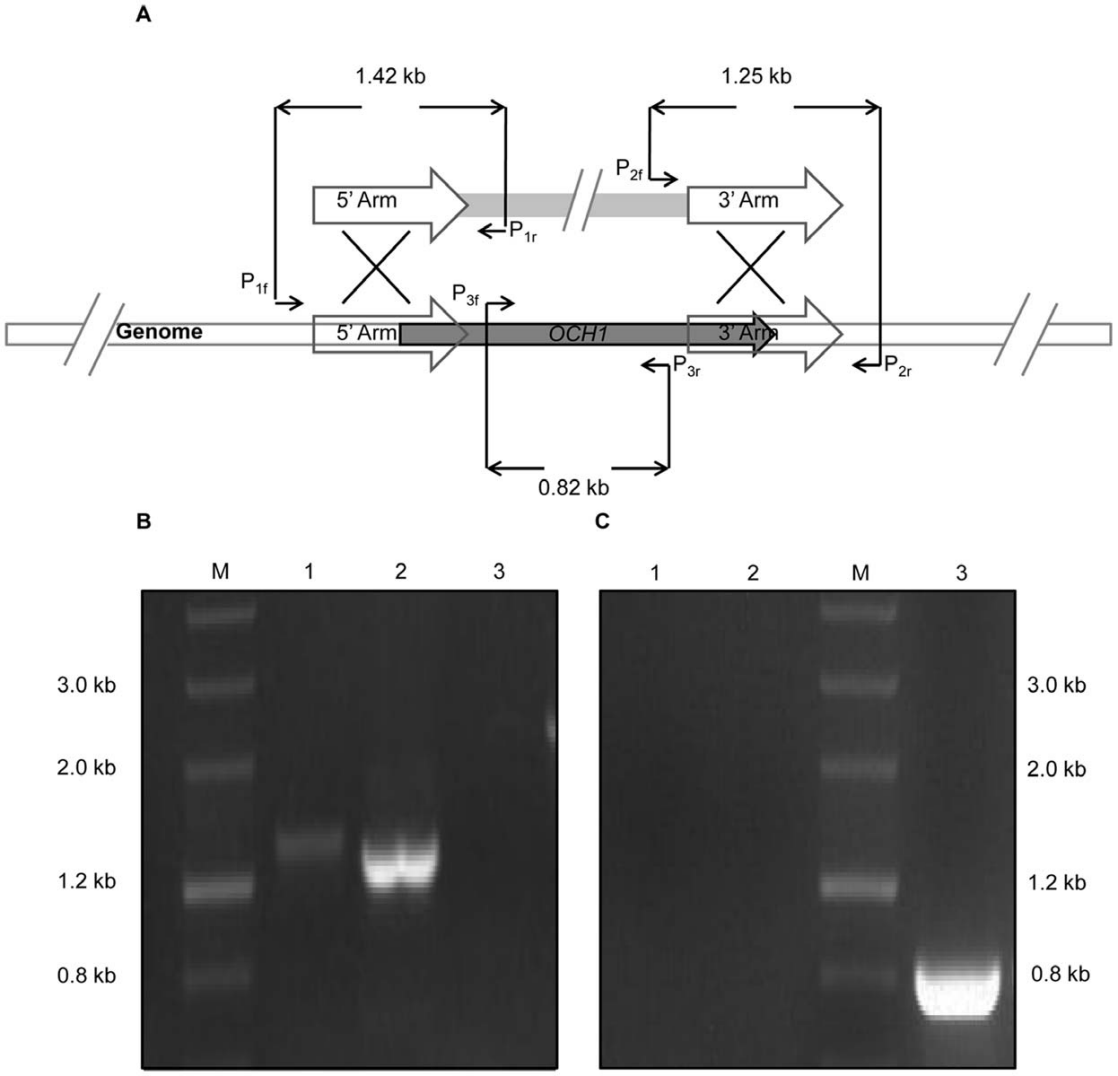
Verification of *och1* mutant by polymerase chain reaction (PCR). A) Schematic representation of the *OCH1* gene disruption and the primers used for verification. Three primer pairs were used. For the correct transformants, two bands with the size of 1.42 and 1.25 kb were amplified using the P_1f_/P_1r_ and P_2f_/P_2r_ primer pairs, respectively. No band was amplified using the P_3f_/P_3r_ primer pair. B) Identification of *och1* mutant by PCR with three primer pairs, P_1f_/P_1r_ (Lane 1), P_2f_/P_2r_,(Lane 2), and P_3f_/P_3r_ (Lane 3). C) *Pichia pastoris* GS115 strain was used as a negative control for PCR verification by using the three primer pairs, P_1f_/P_1r_ (Lane 1), P_2f_/P_2r_ (Lane 2), and P_3f_/P_3r_ (Lane 3; the expected size of the band is 0.82 kb).

The transformants were further analyzed by colony PCR to verify whether they contained the correct chromosomal integrations of the gene disruption cassette. The parental strains were used as the control group. Two primer pairs (Fig. 4A), P_1f_ (located upstream of the 5′ homologous region in the genome)/P_1r_ (located within pZeoloxp) and P_2f_ (located within pZeoloxp)/P_2r_ (located downstream of the 3′ homologous region in the genome), were used to verify each gene. The successful amplification of both bands with the expected size indicated that the chromosomal integrations were correct. The amplification of one band with either primer pairs corresponded to single crossover recombination. The P_3f_/P_3r_ primer pair (Fig. 4A), located within the open reading frame, was used for further verification, and no band should be amplified for the correct disruptants.

### New Disruption Method and Verification

A typical process involves three steps. First, the helper plasmid, such as pGKARSmazf-OCH1, was introduced into the GS115 strain to generate the transition host, GS-tranOCH1. The cells were screened on the YPD medium with 500 mg L^–1^ of G418. The transformants that harbor the episomal expression plasmids were confirmed by performing colony PCR with the P_5′GAP_/P_3′AOX1_ and P_5′AOX1_/P_3′AOX1_ primer pairs. Second, the transition host was subjected to a one-step disruption method. Third, the helper plasmid was removed by streaking on MM agar plates [13.4 g L^–1^ of YNB, 0.5% (v/v) methanol, 0.1 g L^–1^ of histidine, 0.04 g L^–1^ of Zeocin, and 0.0004 g L^–1^ of biotin]. The P_5′GAP_/P_3′AOX1_ and P_5′AOX1_/P_3′AOX1_ primer pairs were used to verify the removal of episomal expression plasmid.

## Results

### Direct Disruption of *OCH1* Gene

α-1,6-Mannosyltransferase, the product of *OCH1* gene, adds the first α-1,6-mannose to the Man9ClcNAc2 core oligosaccharide and initiates several subsequent high mannose-type N-glycosylations [18]. Accordingly, *OCH1* is often chosen as the deletion target to avoid the hyperglycosylation of the expressed heterologous proteins in yeasts. However, *OCH1* deletion is a very inefficient process according to previous studies. Choi and his colleagues [9] obtained only one *P. pastoris* strain with the inactivated *OCH1* gene from approximately 1000 clones using double homologous strategy with 2878/1011 bp length of homologous flanks. Using homologous flanks as long as 3 kb, Vervecken et al. [15] failed to delete the *OCH1* gene following the same strategy. To test the efficiency of *OCH1* deletion, we first used the direct one-step knockout method, and the results were used as a control group for the succeeding experiment.

We initially constructed a disruption vector for the *OCH1* gene inactivation. This vector was generated by inserting two flanking regions, which were amplified from the *P. pastoris* GS115 genome, into the pZeoloxp module plasmid by performing a three-fragment ligation. The two homologous flanks, 5′ and 3′ regions of the disruption target, were 874 and 852 bp, respectively. The disruption plasmid, designated as pZeoloxp-OCH1, was then linearized at the *Not*I site and transformed into *P. pastoris*. More than 1000 clones were initially analyzed by PCR using the P_1f_/P_1r_ and P_2f_/P_2r_ primer pairs (Fig. 4A). Only one clone, designated as KO24#, showed one expected amplified fragment (1.42 kb). However, the other expected 1.25 kb fragment was not amplified from KO24#, suggesting that KO24# may be the result of a single crossover recombinant event at the 5′ homologous region.

### Construction of Plasmid pGKARSmazf-OCH1 for Modification of *P. pastoris*

To apply our proposed strategy, we initially provided a backup *OCH1* gene for *P. pastoris* before deletion to avoid compromising the fitness of the yeast cells because of function loss. This process was performed by cloning *OCH1* into a carefully designed episomal pGKARSmazf-OCH1 vector (Fig. 3), which is characterized by three elements: 1) *Pichia* ARS2 (PARS2) fragment, which kept the plasmid inside the cells for a considerable time and allowed the easy removal when needed; 2) the strong constitutive glyceraldehyde-3-phosphate dehydrogenase (GAP) promoter; and 3) the *mazf* expression cassette regulated by promoter AOX1, whose presence is essential for plasmid removal (as explained later).

The pGKARSmazf-OCH1 plasmid was introduced into the GS115 strain by electroporation without linearization. The transformants were screened on YPD plate that contains 500 mg L^–1^ of G418. Approximately 195 colonies were formed per µg of DNA. Colony PCR was performed using the two primer pairs, P_5′GAP_/P_3′AOX1_ and P_5′AOX1_/P_3′AOX1_, to verify the positive clones. Among the 16 clones, 15 showed the two expected bands, particularly 618 and 1837 bp for *mazf* and *OCH1* cassettes, respectively (data not shown).

### Disruption of the Chromosomal *OCH1* Gene and Elimination of the pGKARSmazf-OCH1 Plasmid

The transition host that carries the redundant *OCH1* copies (designated as GS-tranOCH1) was used as the host for the *OCH1* gene disruption. GS-tranOCH1 was transformed using the linearized pZeoloxp-OCH1 disruption plasmid based on the same procedure used in the direct one-step method. Approximately 60 transformants per µg of DNA were obtained, randomly picked, and analyzed by colony PCR using the P_1f_/P_1r_ and P_2f_/P_2r_ primer pairs. The results showed that the expected 1.42 kb band was amplified by the P_1f_/P_1r_ primer pair from six clones, which were further analyzed using the P_2f_/P_2r_ primer pair. All of the clones also revealed the expected 1.25 kb band (data not shown), suggesting that the chromosomal *OCH1* was successfully deleted by gene replacement. The results were also confirmed by sequencing the 1.42 and 1.25 kb PCR products.

To remove pGKARSmazf-OCH1, *och1* mutant transition strain (designated as GS-tranOCH1-ΔOCH1) was streaked on a MM plate, in which the *mazf* could be expressed with the AOX1 promoter induced by methanol in the medium. The *mazf* gene, which was obtained from *E. coli*, encodes the MazF toxin that functions as an mRNA interferase and inhibits the growth of prokaryotes and eukaryotes [19], [20]. Therefore, *mazf* expression likely causes a strong selection pressure on streaked strains and forces them to lose the obtained plasmids. The colonies, which were much smaller than that of the GS115 strain, appeared after the strain was cultured for 3 d at 30°C. A change in the colony morphology from smooth to rough appearance was also observed, which was consistent with the previous characterizations of *och1* mutant strains [21]. Ten randomly selected colonies on the MM plate were determined by colony PCR with the P_1f_/P_1r_, P_2f_/P_2r_ and P_3f_/P_3r_ primer pairs to verify *OCH1* deletion and plasmid clearance. All of the 10 clones exhibited the desired amplification pattern (Fig. 4B), in contrast to control (Fig. 4C), suggesting the efficient removal of helper pGKARSmazf-OCH1 plasmid from *och1* mutant strains (resultant strains were named GS-ΔOCH1).

**Figure 5:**
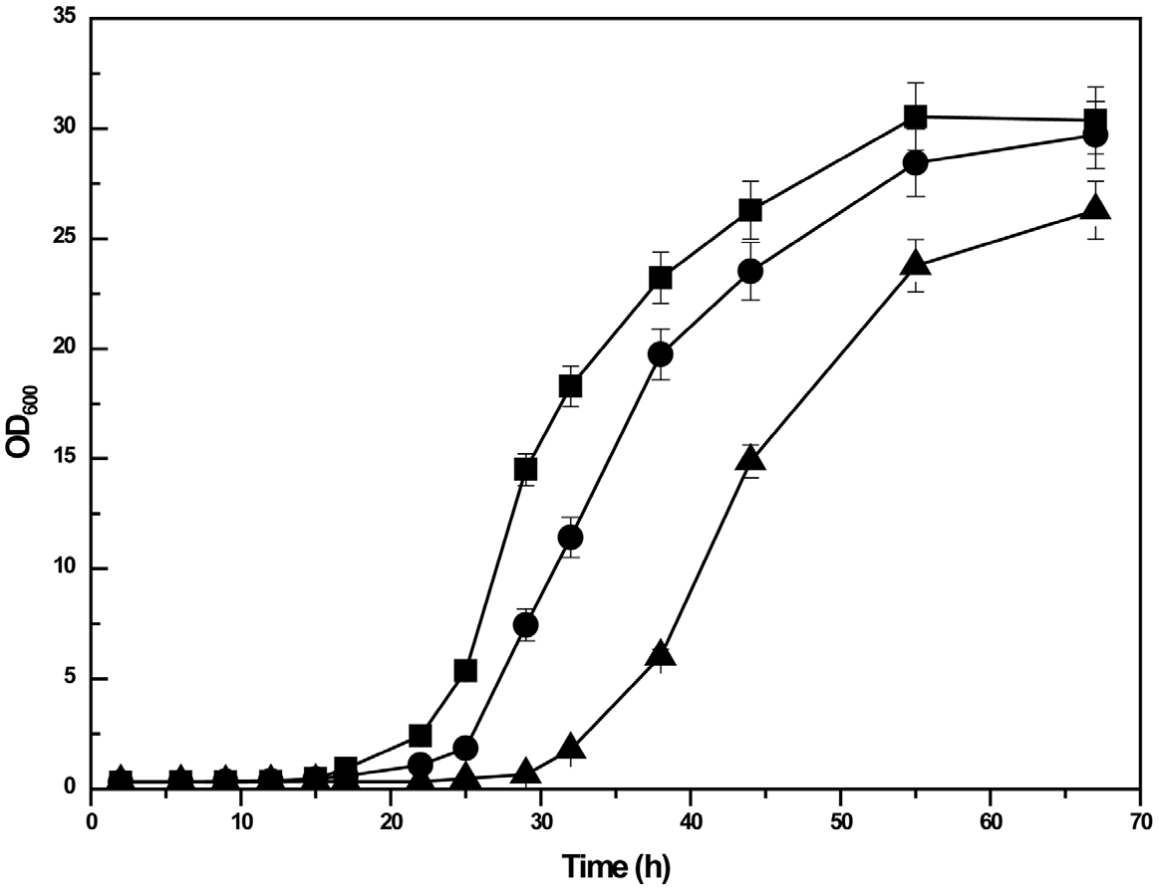
Comparison of cell growth of GS115 (shaded square), GS-tranOCH1-ΔOCH1 (shaded circle), and GS-ΔOCH1 (shaded triangle). Biological triplicate cultures of all three *P. pastoris* strains are grown at 30°C with 200 rpm shaking in 50 ml Falcon tubes containing 5 ml of YPD medium. Turbidity is monitored at OD_600_.

The growth profiles of GS115, GS-tranOCH1-ΔOCH1, and GS-ΔOCH1 were then compared. The results showed that the duration of the lag phase of GS-ΔOCH1 was approximately doubled compared with that of GS115 strain, indicating that the loss of the *OCH1* gene is severely detrimental to yeast growth (Fig. 5). By contrast, the lag phase of GS-tranOCH1-ΔOCH1 was slightly longer than that of GS115, suggesting that the *och1* phenotype can be rescued by the presence of the redundant *OCH1* gene in the episomal expression plasmid to a large extent.

### Disruption of the Chromosomal *SGS1* and *KU70* Genes

To assess the effectiveness of our strategy, two *P. pastoris* genes, namely, *SGS1* and *KU70* were selected for gene-targeted deletion. The conventional one-step strategy and the new strategy were both applied. The disruption plasmids for *SGS1* and *KU70*, namely, pZeoloxp-SGS1 and pZeoloxp-KU70, as well as two transition strains with the redundant copies of the corresponding genes, were generated based on the same procedure used in the *OCH1* gene. Table 2 shows that the *KU70* deletion by the one-step strategy resulted in five positive clones among the 34 selected transformants, whereas ten positive clones were obtained by performing the new strategy, indicating a 1-fold increase in the frequency of *KU70* gene targeting. For *SGS1*, the frequency of obtaining positive clones was increased from 1% to 24% (Table 2), which is a 23-fold increase, compared with that in one-step strategy.

**Table 2:** Comparison of gene targeting efficiencies between conventional and new strategies.

Recipient strain	Length of flankings (bp)(Left/Right)	Correct disruptants^a^	Total clones	Frequency of targeting^b^
***OCH1*** ** gene deletion**				
**GS115**	874/852	0	1000	0%
**GS-tranOCH1**	874/852	6	60	10%
***KU70*** ** gene deletion**				
**GS115**	911/976	5	34	15%
**GS-tranKU70**	911/976	10	34	30%
***SGS1*** ** gene deletion**				
**GS115**	1011/988	1	103	1%
**GS-tranSGS1**	1011/988	8	34	24%

a Correct disrupants are clones with correct gene targeting (*i.e.* gene replacement by homologous recombination) by PCR verification;

b Frequency of targeting is defined as the ratio of number of correct disrupants to the number of total examined clones by PCR verification.

## Discussion

For non-conventional yeasts, efficient gene targeting remains a challenge, which largely limits the study and the application of these industrially important strains. To address this issue, two strategies were most commonly employed: 1) increasing the HR efficiency, usually by increasing the homologous arm length. However, longer flanks are not always sufficient for a high percentage of homologous integration [22], [23]; 2) suppressing the NHEJ pathway by deleting important functional proteins, such as KU70 and KU80, involved in the NHEJ pathway. For instance, the deletion of the *KU70* gene in *K. marxianus* yields 80% homologous gene targeting efficiency by using homologous sequences with at least 40 bp in length [24]. The integration at *HIS4* and *ADE1* loci results in >90% targeting efficiencies with only 250 bp of flanking homologous DNA when the *KU70* homolog of *P. pastoris* is knocked out [25]. Nevertheless, the stability and robustness of these strains should be further evaluated.

While previous studies have mainly focused on increasing the HR efficiency or decreasing the competition from the NHEJ pathway, one fact that is overlooked is that the strains after gene deletion on molecular and cellular basis, require a recovery and proliferation process to form colonies. Unfortunately, several non-conventional yeasts, such as *P. pastoris*, *H. polymorpha*, and *K. lactis*, are predominantly haploid. The deletion of functionally important genes, particularly when these genes do not have paralogs in the genome [26], often results in the loss of fitness, which inhibits the proliferation into sizeable colonies. This condition may be more evident under unfavorable conditions, such as cellular recovery from electroporation and growth on solid media with poor nutrition. For instance, the *acs2* mutant of *K. lactis* requires three weeks to form large colonies [27]. Therefore, the possibility that the actual gene targeting efficiency can be reduced is high because of the unformed colonies.

Based on this rationale, we aimed to improve the fitness of haploid yeast cells by providing backup genes presented in a well-designed episomal vector. The fitness of mutant cells was improved effectively by this strategy. The growth defect of *och1* mutant cells was restored to a large extent. As a result, the improvement of gene targeting efficiencies with this strategy is significant. The *och1* disruptants, which cannot otherwise be acquired in practice by direct one-step deletion [15], can be obtained at a frequency of approximately 10% with mediate length of homologous flanks. The efficiencies of the other two genes, *KU70* and *SGS1*, were also increased by 1- and 23-fold, respectively. These results validated our assumption that cellular fitness is an important factor that limits the efficiency of gene targeting in non-conventional yeasts.

In summary, we provided an efficient gene targeting strategy for non-conventional yeasts. The targeted gene was initially amplified by cloning into a helper expression plasmid. After the gene was successfully deleted from the genome, the helper plasmid was removed. Thus, the *och1* disruptants were obtained at a frequency of 10%. The gene targeting efficiencies of *SGS1* and *KU70* were increased by 1- and 23-fold, respectively.

## Supporting Information

Table S1 Primers used in this study.
(DOC)
