# Supplementary material for: Enhancement of the Gene Targeting Efficiency of Non-Conventional Yeasts by Increasing Genetic Redundancy
Source: PLoS One. 2013 Mar 7;8(3):e57952. doi: 10.1371/journal.pone.0057952 (PMC3591452; doi:10.1371/journal.pone.0057952)
Supplement: Table S1 — Primers used in this study. (DOC) [file pone.0057952.s001.doc]

**Table S1. Primers used in this study.**

| **Primers** | **Sequences ( 5’-3’)** | **Source** |
| --- | --- | --- |
| **P_zeo1_** | TGC**AGATCT**CCCACACACCATAGCTTC | This work |
| **P_zeo2_** | CGT**GAGCTC**AGCTTGCAAATTAAAGCC | This work |
| **P_ori1_** | GTC**GTCGAC**CCCGTAGAAAAGATCAAA | This work |
| **P_ori2_** | CTG**GTCGAC**ATGTGAGCAAAAGGCCAG | This work |
| **OCH1-N_5_** | ATTG**GCGGCCGC**TGGTAGGGATGCAATACAAGGT | This work |
| **OCH1-N_3_** | CGCCG**AAGCTT**GGAAAGGAACTATCAGAGGGAGA | This work |
| **OCH1-C_5_** | CGCCG**AAGCTT**ATCGTTGGAAGAACGTGATGCC | This work |
| **OCH1-C_3_** | ATTG**GCGGCCGC**CGGAAAGTGTCAATGGGAAGAG | This work |
| **SGS1-N_5_** | ATTG**GCGGCCGC**ACTCCTGTTTACGCCTGCCA | This work |
| **SGS1-N_3_** | CGCCG**AAGCTT**AACCTTCGCTCGCTTAGCTG | This work |
| **SGS1-C_5_** | CGCCG**AAGCTT**TATTATCAGGAAACAGGGCGAG | This work |
| **SGS1-C_3_** | ATTG**GCGGCCGC**CATTTTGGGGCAGTTTGAATG | This work |
| **KU70-N_5_** | ATTG**GCGGCCGC**AAAGACAGCCTGCTGGAATTG | This work |
| **KU70-N_3_** | CGCCG**AAGCTT**ACACCCTATTCCTGTACCGGG | This work |
| **KU70-C_5_** | CGCCG**AAGCTT**CACGGCTTGCCAACACTCTC | This work |
| **KU70-C_3_** | ATTG**GCGGCCGC**TGGTTATTCCCTTCCCTGGC | This work |
| **P_kan1_** | ATTG**CCATGG**GCATGAGCCATATTCAACGGGA | This work |
| **P_kan2_** | CGCCG**AGGCCT**TTAGAAAAACTCATCGAGCA | This work |
| **P_pars2-F_** | CATC**GGATCC**TCGAACATAGTCCGTCCCCG | This work |
| **P_pars2-R_** | CAAG**AGATCT**TCGATGTCGACTCAACCTAT | This work |
| **OCH1-F** | ATTG**GAATTC**CTCTATCGCTCTCCTTCCAAGT | This work |
| **OCH1-R** | CGCCG**CATATG**CTATGATGACGGACGATCGCT | This work |
| **SGS1-F** | ATTG**GAATTC**TAATCAACCTTTTCTCCGCA | This work |
| **SGS1-R** | CGCCG**CATATG**CTACCACGATGATACTGTTT | This work |
| **KU70-F** | ATTG**GAATTC**TCACATCTCTTCTTCTCACA | This work |
| **KU70-R** | CGCCG**CATATG**CATTGCATTACAACATAGGC | This work |
| **P_mazf1_** | ATTG**GAATTC**ATGGTAAGCCGATACGTACC | This work |
| **P_mazf2_** | CGCCG**GCGGCCGC**CTACCCAATCAGTACGTTAA | This work |
| **P_1f_** | TAGCGGCACTTCCTGCTACT | This work |
| **P_1r_** | TGTGCTTGGGTGTTTTGAAG | This work |
| **P_2f_** | GACGACGTGACCCTGTTCATC | This work |
| **P_2r_** | GATCTTCTTCGGGAGTGAAC | This work |
| **P_3f_** | GAAGGCAGATGGCAGTTTGCTCTAC | This work |
| **P_3r_** | ACTTCCACGATCCTTTCCTTCCAC | This work |
| **P_1f_-SGS1** | GTCCGAGCTAATGATTGCTA | This work |
| **P_2r_-SGS1** | GATGAGGGTTCATACCTGCC | This work |
| **P_1f_-KU70** | GACAAGGTCATAGGATCTGA | This work |
| **P_2r_-KU70** | GCAGAGTGCCAAATGAACGA | This work |
| **P_3f_-SGS1** | TGTCTCCCACTCAACCACAA | This work |
| **P_3r_-SGS1** | CTCCTCCTGTTGGCATCAAA | This work |
| **P_3f_-KU70** | CAGTAAACCGTTATCAGCCA | This work |
| **P_3r_-KU70** | TGGCGTACATCATCCAGATA | This work |
| **P_5’GAP_** | GTCCCTATTTCAATCAATTGAAC | This work |
| **P_5’AOX1_** | GACTGGTTCCAATTGACAAGC | This work |
| **P_3’AOX1_** | GGCAAATGGCATTCTGACATCC | This work |

Restriction sites are in bold font.
